# Hip pain in children with cerebral palsy: a population-based registry study of risk factors

**DOI:** 10.1186/s12891-019-2449-8

**Published:** 2019-02-08

**Authors:** Alexander Marcström, Gunnar Hägglund, Ann I. Alriksson-Schmidt

**Affiliations:** Lund University, Skåne University Hospital, Department of Clinical Sciences Lund, Orthopedics, Lund, Sweden

**Keywords:** Cerebral palsy, Hip, Pain, Register, Risk factors

## Abstract

**Background:**

Hip pain is prevalent in children with cerebral palsy (CP). Hip displacement is a known risk factor for hip pain. However, many children do not have displaced hips but still have hip pain and the aetiologies are poorly understood. The aims of this study were to investigate: 1. the prevalence of hip pain related to age, gender, gross motor function, degree of hip displacement and 2. the associations between hip pain and age, gender, gross motor function, degree of hip displacement, ranges of hip and knee motion (ROM) and degree of spasticity in the muscles around the hip.

**Methods:**

This was a cross-sectional retrospective register study based on data from the Swedish follow-up programme and national healthcare registry CPUP, which includes > 95% of children with CP in Sweden. The participants were born in 2000 or later and 4–16 years of age. Data from the latest examination were used. In Aim 1, the prevalence of hip pain was calculated using frequencies and crosstabs. Differences between groups were calculated using chi-square tests and independent samples t-tests. In Aim 2, associations between hip pain and the variables were analysed using logistic regression.

**Results:**

The overall prevalence of hip pain was 7%. No significant gender difference was found. Hip pain prevalence increased with age, lower gross motor function and higher degree of hip displacement. The median migration percentage (MP) in painful hips was 26%, compared to 21% in hips where pain was not reported. In the multivariable analysis, significant associations with hip pain were found for MP > 30% and decreased ROM in abduction, flexion and inwards rotation of the hip (*p* < 0.05).

**Conclusion:**

Hip displacement was associated with hip pain. However, hip displacement was not present in the majority of painful hips. In addition to hip displacement, decreased ROM was also associated with hip pain.

**Electronic supplementary material:**

The online version of this article (10.1186/s12891-019-2449-8) contains supplementary material, which is available to authorized users.

## Background

Cerebral palsy (CP) is a heterogeneous group of permanent disorders of movement and posture appearing in early childhood. The severity, type and site of the movement and posture impairments vary widely. CP is often, especially in more severe cases, accompanied by additional impairments such as those of communication, sensation or cognition [1]. Secondary conditions, which per definition are preventable, are also common [2]. Pain is one of the more frequent secondary conditions in CP. A systematic review showed that three of four children with CP were in pain [3]. Pain has been found to reduce both self-reported quality of life and participation in life situations [4, 5].

Gross motor function is often described with the gross motor function classification system (GMFCS), consisting of five levels, where level V describes the most impaired gross motor function [6]. There are conflicting results regarding the association between pain and gross motor function. Some studies report no association [4, 7], while others report higher frequency of pain in children with lower gross motor function [8, 9]. In a recent registry study on pain including 2777 children with CP in Sweden, pain sites differed according to the GMFCS. Pain in the feet was most commonly reported at GMFCS I, knee pain at GMFCS III and abdominal and hip pain at GMFCS V [10]. Several studies have found that girls with CP report pain more frequently than boys, which is in line with gender differences found in the general population [9–11]. In addition, the frequency of pain generally increases significantly with age in this population. [9] Spasticity and decreased range of motion (ROM) are other factors that have been associated with pain in individuals with CP [12, 13].

One of the most common pain sites in children with CP is the hips [10]. The aetiologies of hip pain are not clear, yet some factors are known to be related. The majority of children with severely displaced or dislocated hips experience hip pain [14]. The risk of developing hip displacements and dislocations increases with lower gross motor function [15], and hip pain is also more frequently reported in children with lower gross motor function (i.e., higher GMFCS level) [10]. Displacements and dislocations of the hips can largely be prevented [16, 17]. Since the introduction of hip surveillance in Sweden in 1994, the incidence of hip dislocations in children with CP has decreased from 8% to a stable 0.4% [17]. However, despite progress in preventing hip dislocations, 19% of children with the lowest gross motor function and 9% of all children with CP at any GMFCS level experience hip pain in Sweden, making it the second most common location of pain, after pain in the feet [10].

The aims of this study were to investigate: 1. The prevalence of hip pain related to age, gender, GMFCS level and degree of hip displacement. 2. The associations between hip pain and age, gender, GMFCS level, degree of hip displacement, ranges of passive hip and knee motion and degree of spasticity in different muscle groups around the hip.

## Methods

This was a cross-sectional retrospective cohort study based on registry data from January 2015 – October 2017.

### Procedure and participants

**Fig. 1:**
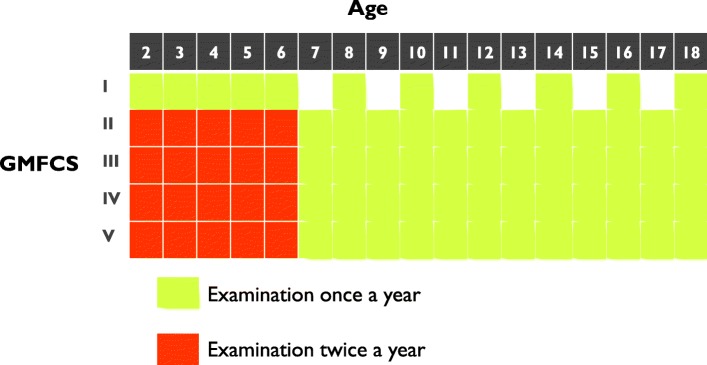
Clinical examination schedule in the Swedish surveillance programme for individuals with CP (CPUP). GMFCS = Gross Motor Function Classification System

This study was based on data from the Cerebral Palsy Follow-Up Programme (CPUP). CPUP is a combined multidisciplinary longitudinal follow-up programme and registry for individuals with CP. Since 2005, the registry is classified as a Swedish national quality registry by the Swedish government, including 95% of children with CP in Sweden born after 2000 [18]. Children with suspected or confirmed diagnoses of CP are invited to participate in CPUP. Before deciding to participate, the legal caregivers are informed that it is possible to withdraw their child from the registry at any time, and that doing so will not affect their children’s healthcare. They are also informed that it is possible to partake in the follow-up program only, and not in the registry. Verbal consent is obtained by the legal caregivers who agree to their children’s participation. By the age of four years, a neuropaediatrician either confirms or dismisses the CP diagnosis. If the diagnosis is dismissed, the child’s data are deleted from the registry. The children with confirmed diagnoses continue to be regularly assessed using a standardized protocol by physiotherapists (PT) and occupational therapists at the habilitation units, and the data are electronically entered into the registry. Depending on the age and the gross motor function, the examination schedule varies from two times per year to once every second year (Fig. 1). This study included data from the latest assessment for children in CPUP aged 4–16 years and born after 2000. Children with baclofen pumps and/or missing data on pain variables were excluded.

The study was approved by the Ethics Board at Lund University (LU 443–99, revised 2009).

### Measures

Age was measured as a continuous variable (whole years) and gender as a dichotomous variable. Gross motor function was measured using the GMFCS Expanded & Revised Version (referred to as GMFCS in this report), which replaced the original GMFCS system in 2007 and expanded the age range to also include 12- to 18-year-olds [6]. The GMFCS describes the child’s gross motor function on a 5-level ordinal scale, where level I indicates the highest and level V the lowest gross motor function. The GMFCS has been shown to have good inter−/intra-rater reliability and stability [19–21]; and is also valid in terms of describing actual motor ability, e.g., it correlates well with ability in activities of daily living [12].

**Fig. 2:**
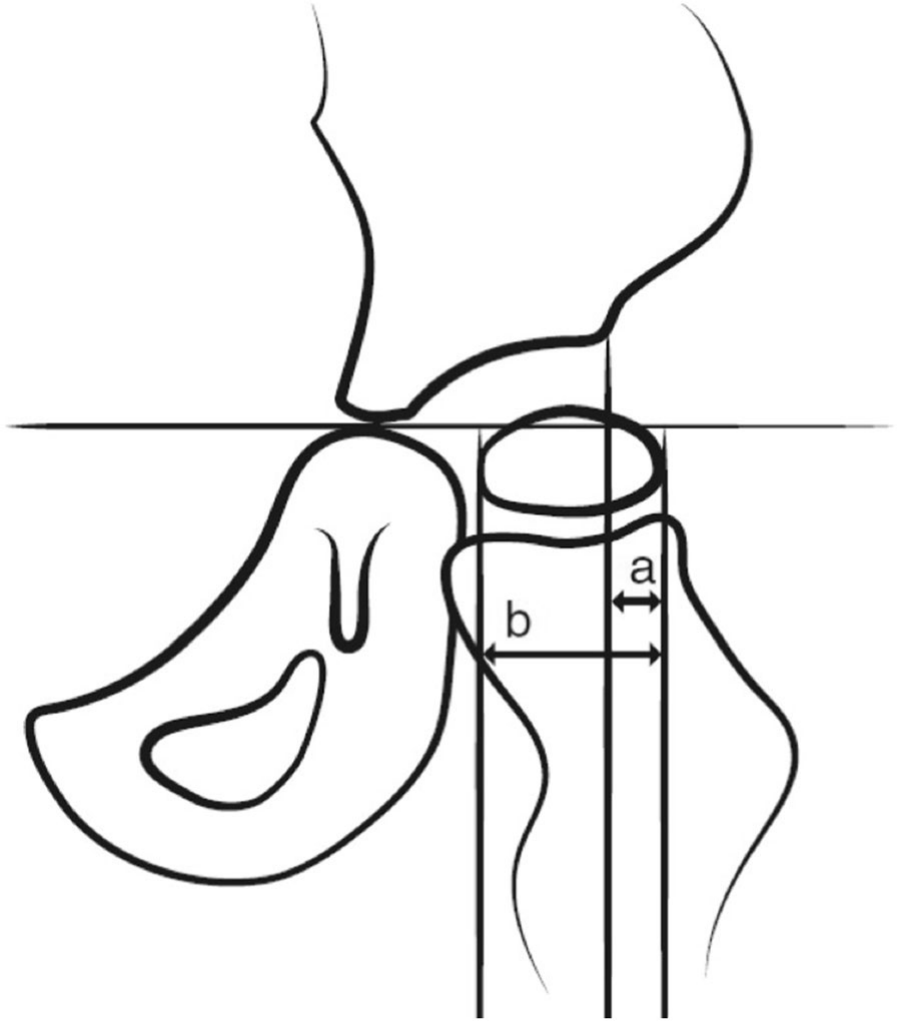
Measurement of the hip migration percentage (MP). MP = a / b × 100. GMFCS = Gross Motor Function Classification System

**Fig. 3:**
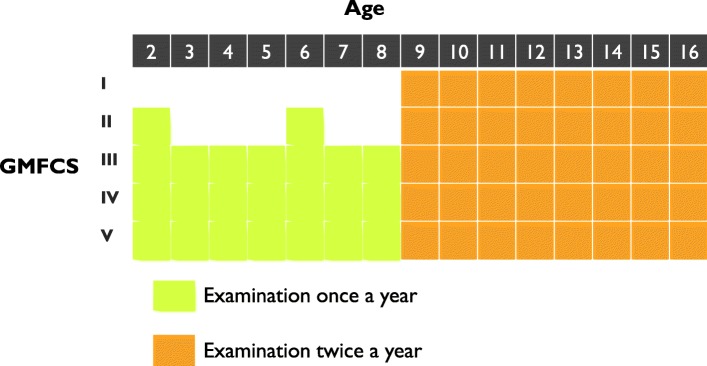
Radiographic examination schedule in the Swedish surveillance programme for individuals with CP (CPUP). GMFCS = Gross Motor Function Classification System ROM = Range of motion

The degree of hip displacement was measured on radiographs with the migration percentage (MP) [22]. The MP ranges from 0 to 100% and measures the proportion of the femoral head positioned lateral to the acetabular margin (Fig. 2). At 100%, the hip is dislocated. In CPUP, children at GMFCS II-V undergo regular radiographic hip examinations, usually once a year (Fig. 3). For children at GMFCS I, radiographic hip examinations are performed only in children with abnormal values at the PT examinations or if the child experiences hip pain***.***

Degree of spasticity was classified with the Modified Ashworth Scale (MAS), a 6-level ordinal scale ranging from 0 to 4 [23]. Moderate to high inter- and intra-rater reliabilities were reported in a recent meta-analysis, with higher reliability for upper than lower extremities [24]. For the purpose of this study, those with scores of 1 or 1+ were combined into one group. Degrees of spasticity in the following five muscle groups were included: Hip flexors, hip extensors, hip adductors, knee flexors and knee extensors. ROM was measured as a continuous variable with goniometer in a standardised position according to the CPUP-manual [25]. The ROM values were rounded to the closest multiple of five and analysed in seven ranges: hip flexion, hip extension, hip abduction, knee extension with the hip straight, knee extension with the hip in 90° flexion and inwards and outwards rotation of the hip. The presence of pain was recorded as a dichotomous variable (pain/no pain). During the PT examination, the child or caregiver (proxy) was asked “are you or do you know/think that the child is in pain?”. If yes, checkboxes were ticked for ten different body locations to indicate the pain site. Since 2016, it is also recorded whether the pain items were self- or proxy reported. If all pain items were left blank, the child was excluded from analysis.

Information on previous hip surgeries were recorded and classified into three groups: (I) Soft tissue surgery (adductor - psoas lengthening), (II) femur osteotomy and (III) pelvic osteotomy. It should be noted that soft tissue surgery could be performed by itself (coded I), however femur osteotomy (coded as II) was always performed in combination with soft tissue surgery, and pelvis osteotomy (coded as III), in turn, was always performed in combination with soft tissue surgery and femur osteotomy. Administration of spasticity-reducing botulinum toxin A in the lower extremities since the latest examination was recorded as yes or no.

## Statistical analysis

It is not recorded in CPUP if the pain was in the right, left or both hips. If assuming pain in both hips and including both in the analyses, the assumption of independence between observations would be violated [26]. Therefore, for every measure, data from only one hip per participant was used. This was done systematically; for each participant and for each measure, the most spastic muscle group, the lowest recorded ROM and the highest recorded MP were used. An alternative would have been to use the mean value of both hips in all participants. This was considered a less ideal option given the large differences between sides in children with unilateral spastic CP.

In Aim 1, the prevalence of pain was calculated using frequencies and crosstabs. Chi-squares and independent-samples t-test were used to assess differences between groups. In Aim 2, binomial logistic regressions were employed to evaluate the associations between the dependent variable (hip pain) and the independent variables. Linear regression was used to test for multicollinearity, with all variables having lower variance inflation factors than 2.0. All variables were linear to the logit of hip pain and were therefore included. All studentized residuals were kept in the analyses. Nagelkerke R^2^ was used to describe the variance in hip pain explained by the model. Listwise deletion was used, meaning that only children with values for all variables were included in the logistic regression. Children with missing data on spasticity, ROM or hip displacement were therefore excluded. Due to small cell sizes, MAS levels 3 and 4 were combined, and MPs were grouped accordingly: 0–30%, 31–40% and 41–100%. Because the indication for radiographic examinations of the hips differ between GMFCS I and the other levels, children at GMFCS I were not included in the primary logistic regression analysis. To be able to generalize the results to children at all GMFCS levels however, a complementary logistic regression analysis was employed, including children at all GMFCS levels without hip displacement as an independent variable, presented in Additional files. Significance was considered at 0.05% level. IBM SPSS Statistics version 24 was used for statistical analysis [27].

## Results

**Fig. 4:**
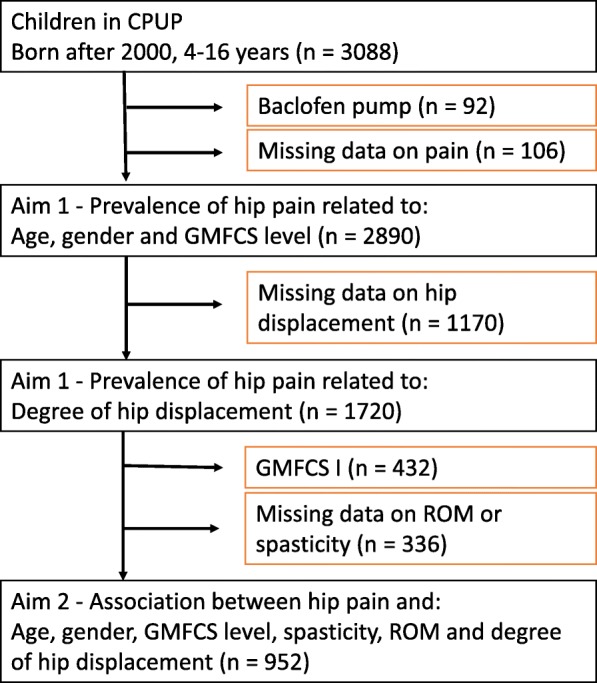
Inclusion flowchart. GMFCS = Gross Motor Function Classification System ROM = Range of motion

A total number of 3088 children met the inclusion criteria. Of these, 198 children had baclofen pumps and/or missing data on pain and were excluded. This did not affect the distribution of gender, age, or GMFCS levels significantly. An inclusion flowchart is presented in Fig. 4. Characteristics of the participants included in the different analyses are shown in Table 1. Pain items were self-reported in 51% of children and proxy reported in 49%. In total, 12%, had undergone soft tissue surgery, femoral osteotomy and/or pelvic osteotomy (GMFCS I = 1%, GMFCS II = 3%, GMFCS III = 14%, GMFCS IV = 27%, and GMFCS V = 46%). Children with higher gross motor function (i.e., lower GMFCS levels) and older ages were more likely to self-report. Characteristics of children who self-reported and those who did not (i.e. proxy reported) are presented in Additional file 1.

**Table 1:** Characteristics of the study population

Characteristics	Aim 1 ^a^	Aim 2 ^b^
Total, n	2890	952
Age, mean (SD)	9.5 (3.6)	9.6 (3.6)
Sex, n (%)		
Boys	1660 (57.4)	572 (60.1)
Girls	1230 (42.6)	380 (39.9)
GMFCS level, n (%)		
I	1327 (45.9)	0 (0.0)
II	489 (16.9)	277 (29.1)
III	266 (9.2)	180 (18.9)
IV	423 (14.6)	290 (30.5)
V	385 (13.3)	205 (21.5)
BTX-A treatment since last assessment, n (%)	563 (19.5)	254 (26.7)
Hip Surgery, n (%)		
Soft tissue	143 (4.9)	83 (8.7)
Femur osteotomy	104 (3.6)	61 (6.4)
Pelvic osteotomy	107 (3.7)	64 (6.7)
Pain, n (%)		
Any site	1127 (39.0)	375 (39.4)
Hip pain	202 (7.0)	86 (9.0)

*GMFCS* Gross Motor Function Classification System

*BTX-A* Botulinum Toxin A

^a^Aim 1: Prevalence analysis

^b^Aim 2: Association analysis

**Fig. 5:**
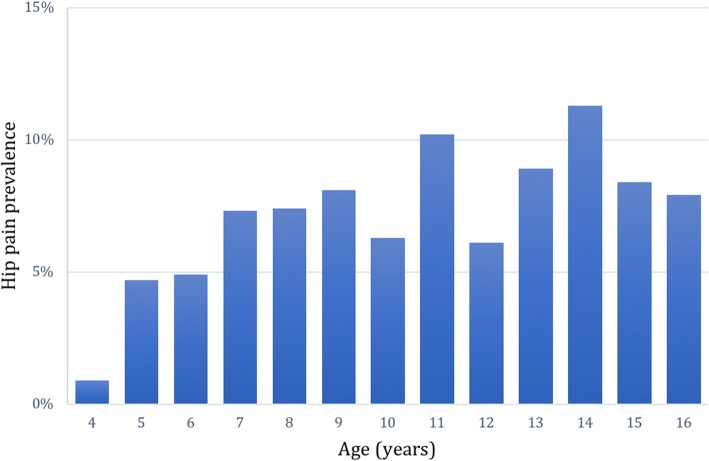
Hip pain prevalence related to age (*n* = 2890)

**Fig. 6:**
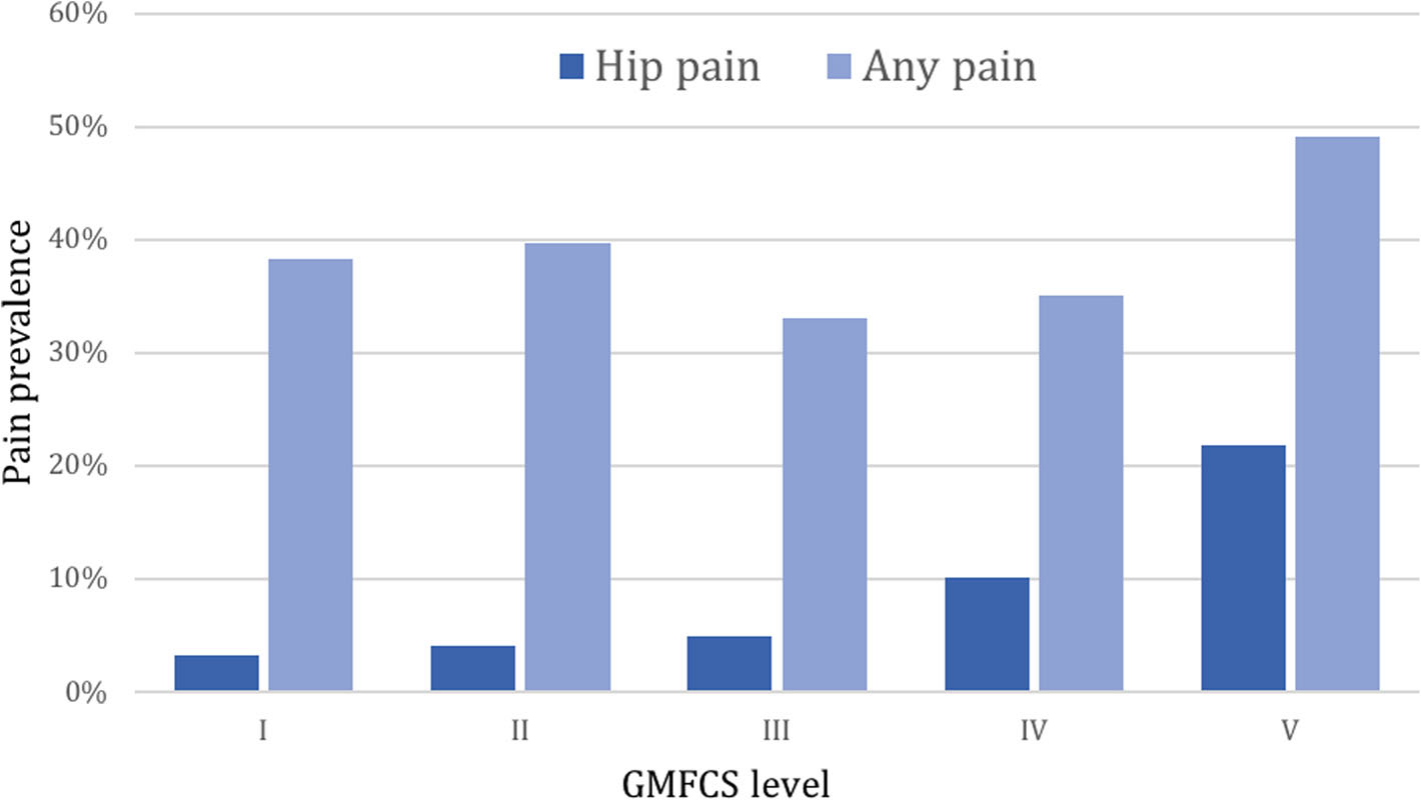
Prevalence of hip pain and pain at any site (including hip pain) related to GMFCS level (*n* = 2890). GMFCS = Gross Motor Function Classification System

**Fig. 7:**
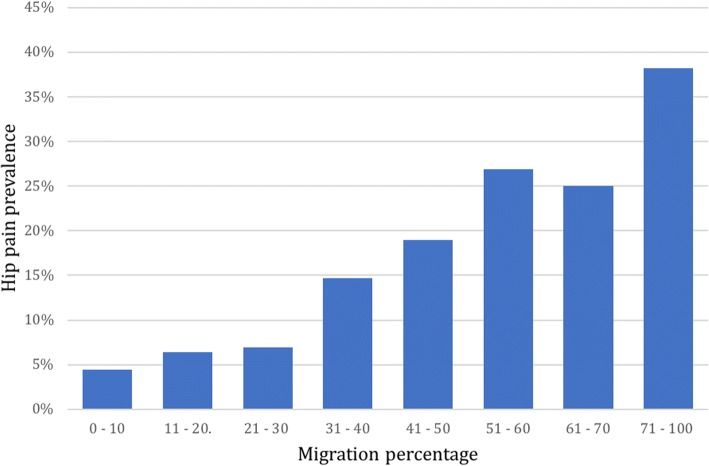
Hip pain prevalence related to hip migration percentage, obtained from latest radiographic examination (*n* = 1720)

In total, 2890 children were included in the prevalence analysis (Aim 1). Pain at any site was reported in 1127 children (39.0%) and hip pain was reported in 202 children (7.0%). In the self-reporting group, 5.3% of the children reported having hip pain (any pain = 40.2%), compared to 8.8% (any pain = 34.7%) in the proxy reported group. The prevalence of hip pain was 7.5% in girls and 6.7% in boys, a non-significant difference. The prevalence increased with age and GMFCS level (Figs. 5 and 6). In the prevalence analysis related to hip displacement, data were available in 1720 children. The hip pain prevalence increased with increasing MP (Fig. 7). The median MP in painful hips was 26%, compared to 21% in hips where pain was not reported.

A total of 952 children were included in the association analysis (Aim 2). The model explained 28% of the variance of hip pain. The results are presented in Table 2. The odds ratio (OR) did not differ significantly with increasing age or for girls compared to boys. Compared to children at GMFCS II, children at GMFCS III and IV did not have significantly higher ORs for hip pain and the OR for children at GMFCS V was 2.40 (95% CI = 0.97–5.96). Compared to spasticity level 0, none of the five muscle groups around the hip had significant ORs on hip pain, except for level 2 in knee extensors. The OR of hip pain was significantly higher for decreased ROM with 5 degrees in three out of seven directions; inward rotation, flexion and abduction of the hip. Compared to MP 0–30%, the OR for hip pain was 2.11 (95% CI = 1.10–4.06) for MP 31–40% and 2.72 (95% CI = 1.32–5.59) for MP 41–100%.

**Table 2:** Logistic regression with hip pain as dependent variable. *N* = 952

Variable	Hip pain / sample size (%)^d^		Odds Ratio (95% CI)	
Age^a^	952		1.0	(0.92 - 1.09)
Gender				
Male	46 / 572	(8.0)	1.0	
Female	40 / 380	(10.5)	1.21	(0.72–2.05)
GMFCS				
II	11 / 277	(4.0)	1.0	
III	10 / 180	(5.6)	1.04	(0.40–2.67)
IV	23 / 290	(7.9)	1.10	(0.46–2.68)
V	42 / 205	(20.5)	2.40	(0.97–5.96)
Spasticity^b^				
Hip flexors level 0	41 / 670	(6.1)	1.0	
level 1	36 / 230	(15.7)	1.50	(0.83–2.72)
level 2	2 / 31	(6.5)	0.54	(0.09–3.25)
levels 3–4	7 / 21	(33.3)	3.55	(0.82 - 15.41)
Hip extensors level 0	40 / 644	(6.2)	1.0	
level 1	33 / 216	(15.3)	1.0	(0.52–1.93)
level 2	8 / 64	(12.5)	0.36	(0.11–1.16)
levels 3–4	5 / 28	(17.9)	0.38	(0.08 - 1.83)
Hip adductors level 0	17 / 385	(4.4)	1.0	
level 1	39 / 387	(10.1)	1.86	(0.91–3.83)
level 2	18 / 128	(14.1)	1.73	(0.69–4.33)
levels 3–4	12 / 52	(23.1)	2.87	(0.91–8.99)
Knee flexors level 0	16 / 275	(5.8)	1.0	
level 1	43 / 458	(9.4)	0.80	(0.38–1.69)
level 2	14 / 156	(9.0)	0.65	(0.26–1.63)
levels 3–4	13 / 63	(20.6)	0.96	(0.30–3.09)
Knee extensors level 0	36 / 592	(6.1)	1.0	
level 1	26 / 265	(9.8)	0.69	(0.36–1.33)
level 2	15 / 65	(23.1)	2.81	(1.18–6.69)
levels 3–4	9 / 30	(30.0)	0.84	(0.20–3.56)
Range of motion^c^				
Knee Extension	952		0.97	(0.85–1.10)
Hamstring	952		0.93	(0.84–1.02)
Hip inward rotation	952		1.11	(1.01–1.20)
Hip outward rotation	952		0.95	(0.88–1.04)
Hip Extension	952		1.09	(0.96–1.25)
Hip Flexion	952		1.18	(1.05–1.32)
Hip Abduction	952		1.20	(1.04–1.41)
Hip migration percentage				
0–30	46 / 721	(6.4)	1.0	1.0
31–40	19 / 147	(12.9)	2.11	(1.1–4.06)
41–100	21 / 84	(25.0)	2.72	(1.32–5.59)

*GMFCS* Gross Motor Function Classification System

*CI* Confidence Interval

^a^Age stated as increase with one year

^b^Spasticity classified according to the Modified Ashworth Scale with levels 3 and 4 grouped together

^c^Range of motion stated as decrease with five degrees

^d^For continuous variables (age and range of motion), only sample size is presented

The additional analysis, with children at all GMFCS levels included (*n* = 2224) and without MP as an independent variable, is presented in Additional file 2. The same pattern as in the main analysis was found for age, gender and spasticity. Children at GMFCS V had significantly higher OR of hip pain than children at level I. The OR were significantly higher for decreased ROM in flexion and abduction of the hip.

## Discussion

We studied the prevalence of hip pain related to age, gender and gross motor function level, and the associations between hip pain and; age, gender, gross motor function level, range of hip and knee motion, spasticity in the muscles around the hip joint and degree of hip displacement. The main findings were that hip displacement and lower range of motion, but not spasticity, were associated with hip pain.

The prevalence of hip pain and pain at any site was similar to other studies based on CPUP data [10, 28], and less than half compared to the population-based SPARCLE study, conducted in seven European countries [9]. This was also the case for the prevalence of pain at any site, which was almost twice as common in the SPARCLE study. In the SPARCLE study, 54% of the children between 8 and 12 years experienced pain in the previous week, [4] a percentage that increased to 74% in a follow up at 13–17 years [9]. Differences in age structure and GMFCS distribution between the two studies might explain some of the differences. Less sensitive pain screening in CPUP, without specified recall time when asking about pain, might also help explain the differences. However, the lower pain prevalence might, to some extent, be contributed to successful prevention of secondary musculoskeletal complications in CPUP [17]. As expected, proxy reports were more common in younger children and for children with lower motor ability. Hip pain was more frequently reported in the proxy reported group than the self-reported group. Part of the reason for this could be that the prevalence of hip pain is higher in children at higher GMFCS levels, and that children reported by proxy generally were at higher GMFCS levels. However, to report on somebody else’s pain is difficult.

There was a trend of increasing prevalence of hip pain with increasing age. However, in the multivariable analysis, no significant association was found. This differs from what is usually observed for pain in this population where pain and age are generally positively associated [13]. Regarding gender, there was no significant difference in hip pain prevalence and no association with hip pain in the multivariable analyses. The lack of age and gender effects on hip pain specifically have been observed previously [14].

The prevalence of hip pain at GMFCS IV and V was higher than at GMFCS I-III. In the multivariable analysis, with children at GMFCS II as reference, only being at GMFCS V was borderline significantly associated with hip pain. In the additional analysis, with children at GMFCS I as reference, being at GMFCS V was significantly associated with hip pain. Possible causes are inactivity, extended periods of time spent in the same position or poor positioning. As the majority in this group did not answer the pain items themselves, it is also possible that the proxy reporters misinterpreted the pain or the pain site, e.g. abdominal pain might have been interpreted as hip pain or vice versa.

There was a trend of increasing prevalence of hip pain with higher MPs. Compared to MP 0–30, the OR for hip pain were significantly higher at MP 31–40% and 41–100%. An association between hip pain and MP > 50% has previously been found in children with CP [14]. In adults with CP, associations between hip pain and MP ≥ 33 has been found [29]. Our study confirms the association between hip pain and higher MPs and implies that the risk for hip pain is increased already from the 31–40 range. In Sweden, surgeries to prevent hip dislocations are sometimes recommended in children with MP > 33%. Our findings do indicate that pain seems to occur at low MPs and that interventions should be considered early to prevent pain. However, the majority of painful hips had MP < 30%, indicating that other factors than hip displacement also drive hip pain.

Decreased ROMs in abduction, flexion and inwards hip rotation of the hip were significantly associated with hip pain. The association between hip pain and decreased ROM has previously been reported in adults with CP [12]. One possible explanation is that decreased ROM causes painful strenuous movement patterns and body positions. It is also possible that children with decreased ROMs are closer to their maximum ROM for longer periods and that this causes pain. However, it is also likely that the maximum ROM is not measured in a painful hip.

Higher spasticity level was not significantly associated with hip pain for any muscle group, except for MAS level 2 in knee extensors. However, wide confidence intervals and absence of significant associations for the other spasticity levels in this muscle group implies that the association could be due to chance and not clinically important. Association between pain and spasticity is not well substantiated in the literature. Children with spastic CP have not been found to report pain more frequently than children with other subtypes [28]. However, spasticity has been related to deterioration in ROM during growth and as such might indirectly affect pain [30].

As this was a cross sectional study, causality cannot be inferred. The pain items in CPUP were created to mainly screen for pain. More information of the pain experience is needed and to that end, more detailed pain items have now been included in CPUP. The lack of data on side of body, type, intensity and duration of pain limited the analysis. Due to some of the children’s comorbidities and/or young age/s, self-report was not always possible. The effect of proxy reports is unclear, in other studies both under- and over reporting of pain have been reported [13, 31]. It should be noted that it is very difficult to know for sure that the pain came from the hip joint even if that is where the pain seemed to originate. It is possible that the pain radiated from other areas, such as the stomach or the spine. Moreover, with respect to generalizability, the rather low MP reported in children with CP in Sweden might limit this to populations with similarly low MPs.

The major strengths of this study come from the large cohort analysed, including almost all children with CP in Sweden at all GMFCS levels. Since almost the entire population of children with CP were included, there is little risk for selection bias and our results are likely generalizable.

## Conclusion

Hip displacement was associated with hip pain. However, hip displacement was not present in the majority of painful hips. In addition to hip displacement, it was found that decreased ROM was associated with hip pain.

## Additional files

Additional file 1: Characteristics of children who self-reported and those who did not (i.e. proxy reported) (DOCX 13 kb)

Additional file 2: Logistic regression, with children at all GMFCS levels included (*n* = 2224) and without MP as an independent variable (DOCX 16 kb)

## Abbreviations

CP: Cerebral palsy
CPUP: Swedish cerebral palsy surveillance program and national quality registry
MAS: Modified Ashworth Scale
MP: Migration percentage
PT: Physiotherapist
ROM: Range of motion
