# Supplementary material for: Hip pain in children with cerebral palsy: a population-based registry study of risk factors
Source: BMC Musculoskelet Disord. 2019 Feb 8;20:62. doi: 10.1186/s12891-019-2449-8 (PMC6368700; doi:10.1186/s12891-019-2449-8)
Supplement: Supplementary file 1 — Characteristics of children who self-reported and those who did not (i.e. proxy reported) (DOCX 13 kb) [file 12891_2019_2449_MOESM1_ESM.docx]

| **Table 3. Characteristics of participants based on self-report and proxy report.** | | | |
| --- | --- | --- | --- |
| **Characteristics** | **Self-Reported** | **Proxy Reported** | **Difference^c^** |
| Gender  Girls | 584 (52) | 545 (48) | χ^2^ = 0.604 (p = 0.44) |
| Boys | 759 (50) | 753 (50) |  |
| GMFCS**^b^** level  I | 815 (70) | 347 (30) | χ^2^ = 480 (p < 0.001) |
| II | 240 (50) | 235 (50) |  |
| III | 121 (47) | 136 (53) |  |
| IV | 141 (35) | 258 (65) |  |
| V | 28 (8) | 332 (92) |  |
| Age, years, mean (SD) | 10.8 (3.2) | 8.2 (3.5) | MD**^d^**: 2,6 (p < 0.001) |
| Total | 1,345 (51) | 1,308 (49) |  |
| **^a^**Numbers are percentages except where otherwise stated.  **^b^**Gross Motor Function Classification System.  **^c^**Chi Square and p-value except where otherwise stated.  **^d^**Mean difference. | | | |
