# Supplementary material for: Hip pain in children with cerebral palsy: a population-based registry study of risk factors
Source: BMC Musculoskelet Disord. 2019 Feb 8;20:62. doi: 10.1186/s12891-019-2449-8 (PMC6368700; doi:10.1186/s12891-019-2449-8)
Supplement: Supplementary file 2 — Logistic regression, with children at all GMFCS levels included (n = 2224) and without MP as an independent variable (DOCX 16 kb) [file 12891_2019_2449_MOESM2_ESM.docx]

| **Additional file 2** Logistic regression with hip pain as dependent variable. N = 2224 | | | | |
| --- | --- | --- | --- | --- |
| **Variable** | **Hip pain / sample size (%)**^d^ | | **Odds Ratio (95% CI)** | |
| Age^a^ | 2224 |  | 0.99 | (0.94 - 1.06) |
| Gender |  |  |  |  |
| Male | 73 / 1297 | (5.6) | 1.0 |  |
| Female | 65 / 927 | (7.0) | 1.39 | (0.96 - 2.03) |
| GMFCS |  |  |  |  |
| I | 38 / 1093 | (3.5) | 1.0 |  |
| II | 15 / 375 | (4.0) | 0.83 | (0.44 - 1.56) |
| III | 10 / 203 | (4.9) | 0.72 | (0.32 - 1.59) |
| IV | 27 / 322 | (8.4) | 1.05 | (0.54 - 2.06) |
| V | 47 / 231 | (20.3) | 2.39 | (1.22 - 4.67) |
| Spasticity^b^ |  |  |  |  |
| Hip flexors level 0 | 83 / 1853 | (4.5) | 1.0 |  |
| level 1 | 42 / 302 | (13.9) | 1.38 | (0.83 - 2.28) |
| level 2 | 4 / 43 | (9.3) | 0.64 | (0.18 - 2.30) |
| levels 3 - 4 | 8 / 26 | (30.8) | 2.12 | (0.58 - 7.74) |
|  |  |  |  |  |
| Hip extensors level 0 | 83 / 1834 | (4.5) | 1.0 |  |
| level 1 | 37 / 278 | (13.3) | 1.06 | (0.61 - 1.84) |
| level 2 | 12 / 78 | (15.4) | 0.57 | (0.22 - 1.44) |
| levels 3 - 4 | 6 / 34 | (17.6) | 0.38 | (0.09 - 1.56) |
|  |  |  |  |  |
| Hip adductors level 0 | 55 / 1457 | (3.8) | 1.0 |  |
| level 1 | 46 / 547 | (8.4) | 1.06 | (0.64 - 1.78) |
| level 2 | 19 / 156 | (12.2) | 0.91 | (0.44 - 1.91) |
| levels 3 - 4 | 16 / 64 | (25.0) | 1.51 | (0.61 - 3.77) |
|  |  |  |  |  |
| Knee flexors level 0 | 38 / 1047 | (3.6) | 1.0 |  |
| level 1 | 63 / 895 | (7.0) | 1.20 | (0.75 - 1.95) |
| level 2 | 19 / 201 | (9.5) | 1.07 | (0.53 - 2.18) |
| levels 3 - 4 | 17 / 81 | (21.0) | 1.71 | (0.70 - 4.20) |
|  |  |  |  |  |
| Knee extensors level 0 | 74 / 1728 | (4.3) | 1.0 |  |
| level 1 | 36 / 382 | (9.4) | 0.95 | (0.57 - 1.59) |
| level 2 | 17 / 78 | (21.8) | 2.81 | (1.33 - 5.94) |
| levels 3 - 4 | 10 / 36 | (27.8) | 1.35 | (0.4 - 4.61) |
| Range of Motion^c^ |  |  |  |  |
| Knee Extension | 2224 |  | 0.96 | (0.87 - 1.05) |
| Hip Extension | 2224 |  | 1.06 | (0.96 - 1.18) |
| Hip Flexion | 2224 |  | 1.14 | (1.05 - 1.23) |
| Hip Abduction | 2224 |  | 1.19 | (1.08 - 1.33) |
|  |  |  |  |  |
| GMFCS = Gross Motor Function Classification System.  CI = Confidence Interval  ^a^Age stated as increase with one year  ^b^Spasticity classified according to the Modified Ashworth Scale with levels 3 and 4 grouped together.  ^c^Range of motion stated as decrease with five degrees.  ^d^For continuous variables (age and range of motion), only sample size is presented.  In this additional analysis, hip migration percentage was excluded as variable. Children at all GMFCS levels could therefore be included. Three variables (ROM in hamstring, inward and outward rotation of the hip) were not linear to the logit of hip pain and were therefore excluded. All studentized residuals were kept in the analysis. | | | | |
